# Perceptions of using lithium in fracture management: a survey of orthopaedic surgeons, fracture patients and the general public

**DOI:** 10.1186/s12891-019-2772-0

**Published:** 2019-08-31

**Authors:** Kathak Vachhani, Cari M. Whyne, Ayal Schaffer, Diane Nam

**Affiliations:** 1 Sunnybrook Research Institute, 2075 Bayview Avenue, MG301, Toronto, ON M4N 3M5 Canada; 2 Department of Surgery, University of Toronto, Toronto, Canada; 3 Institute for Biomaterials and Biomedical Engineering, University of Toronto, Toronto, Canada; 4 Department of Psychiatry, University of Toronto, Toronto, Canada

**Keywords:** Lithium, Fracture healing, Drug repurposing, Orthopaedic surgeons, Surveys and questionnaires, Perception

## Abstract

**Background:**

Lithium, an established psychiatric medication, has recently been shown to enhance new bone formation in preclinical fracture models. Current research is focused on evaluating the efficacy of low-dose, short-term lithium treatment to improve long bone fracture healing through a Phase II randomized clinical trial (LiFT NCT02999022). In working towards future applications of lithium for fracture management, this study aimed to understand the current perceptions of lithium as a psychiatric drug and the potential barriers to its orthopaedic adoption.

**Methods:**

Three questionnaires, evaluating knowledge about lithium and willingness to embrace its use in fracture healing were disseminated among the general population, fracture patients eligible for the LiFT (Lithium for Fracture Treatment) trial and orthopaedic surgeons across Canada.

**Results:**

Of the 768 public respondents, 84% were willing to take a medication that would aid fracture healing but only 62.6% if the medication was lithium. Willingness dropped to 44.6% among the 168 respondents who knew about the psychiatric use of lithium. Lack of sufficient knowledge (*n* = 50) and concerns about side effects including effects on the brain (*n* = 74) were the main reasons cited by those who were unwilling to use lithium. Of the 29 fracture patients, only 20 patients had previously heard of lithium. Of these, 40% were willing to take lithium for fracture healing with an additional 10% if the dose was low or if the intake duration was short. Only 50% knew that lithium has side effects. Of the 43 orthopaedic surgeons, 38 surgeons knew about clinical use of lithium. Of these, 68% knew that lithium has side effects and 29% knew that it interacts with other drugs. While most agreed that new strategies are needed to improve fracture management, only 68% were willing to prescribe lithium for fractures with an additional 16% if there is scientific evidence and/or a standard dosing protocol.

**Conclusions:**

This study identified a lack of knowledge about uses and side effects of lithium among all three cohorts. A robust educational framework for orthopaedic surgeons, their patients and the members of their clinical care teams will be essential to widespread repurposing of lithium for fracture care.

**Electronic supplementary material:**

The online version of this article (10.1186/s12891-019-2772-0) contains supplementary material, which is available to authorized users.

## Background

Fracture healing is a proliferative physiologic process that can fail in up to 10% of cases despite appropriate and timely orthopaedic care [1, 2]. Fracture management, particularly in cases of delayed healing, results in significant costs to the healthcare and the individual in loss of function and work productivity. An inexpensive, non-invasive strategy to predictably increase bone healing and provide earlier mobility and independence is currently not available.

Lithium therapy is well known for the treatment of bipolar disorder. Under proper clinical monitoring, psychiatrists have widely prescribed lithium demonstrating its safe and effective administration over prolonged periods [3]. Recently, a positive link has been established between lithium and enhanced bone repair. Lithium is a glycogen synthase kinase-3β (GSK-3β) inhibitor which activates the canonical Wingless (Wnt)/β-catenin signaling pathway that is of significance in bone biology and as a mechanism of increasing bone formation [4]. Preclinical models have been used to identify the optimal administration parameters for lithium in the context of fracture healing. It was shown that a low dose of oral lithium administered during the transition from soft to hard callus stages of fracture healing demonstrated a 46% increase in strength of healing femoral fractures compared to untreated controls [5, 6].

The strength of these preclinical findings has led to the initiation of a Phase II clinical trial evaluating the effect of lithium treatment (300 mg daily from week 2 to week 4 post fracture injury) on long bone fracture healing by relative stability in otherwise healthy patients (LiFT trial, NCT02999022). This represents a simple regimen at a very low dose of 300 mg, with little risk of systemic side effects such as diarrhea, weight gain and increased thirst that are typically experienced with chronic intake of 900–1800 mg. This is different from the therapeutic dosing range of lithium indicated for its psychotropic benefits where dose dependent side effects occur at a much higher dosing range from 900 to 1800 mg/day with long term use.

In working towards future adoption of lithium for fracture treatment, it is important to understand potential barriers that may hinder its clinical uptake. As such, it is critical to understand the current perceptions of lithium as a psychiatric drug and in its other non-medical uses. The objective of this study was to understand perceptions of the general public, patients, and orthopaedic surgeons towards the use of lithium in the context of fracture treatment and factors affecting the perceptions of these three cohorts.

## Methods

Three questionnaires were designed to evaluate the level of knowledge about lithium and the willingness to consider an alternative use of lithium in fracture healing among three distinct populations: the general population, fracture patients eligible to participate in the LiFT trial and orthopaedic surgeons across Canada. The survey questions fell under three broad categories: background information, knowledge about lithium in fracture repair and opinion on the use of lithium for fracture treatment (Additional file 1). Descriptive statistics are presented separately for the three cohorts. Data has been reported as percentages of the respective group size. Given the exploratory nature of this study, qualitative analysis was performed to identify trends in knowledge and opinions about lithium and its adoption in orthopaedics.

### General population survey

An electronic general population survey was created using SurveyMonkey and disseminated via online platforms. In a series of yes or no questions, each respondent was asked: 1) if they knew about the clinical role of lithium for fracture treatment; 2) if the respondent experienced a fracture, would they be willing to take a medication to improve healing; 3) if the respondent had a fracture, would they be willing to take a medication if it is also prescribed for mental illness, and specifically to take lithium. Where relevant, the respondent was asked to justify their choice in free-text responses.

### Patient and surgeon survey

The patient and surgeon questionnaires were approved by the institutional research ethics board as part of the LiFT trial.

#### Patient questionnaire

All patients who were admitted to our level 1 trauma centre between May 2017 and September 2018, had a long bone fracture, and met the inclusion/exclusion criteria (18–55 years of age, otherwise healthy, injury ≤14 days old) to participate in the LiFT trial were asked to complete the questionnaire. Those who declined to participate in the trial completed the questionnaire during their regularly scheduled visit to the fracture clinic (within 2 weeks of injury), while those who participated in the trial completed the questionnaire at their last follow-up visit (between 4 and 6 months post injury).

In the questionnaire, only those patients who knew about lithium, whether in a clinical or non-clinical context, were asked to answer the following questions. The patient was asked if they would be willing to take lithium to improve fracture healing and if not, whether a low dose or short duration regimen would affect their willingness to participate and change their opinion concerning potential side effects of lithium. Additional questions focused on the possible stigma associated with lithium use as perceived by an individual’s social and professional network such as submitting insurance claims or filling a lithium prescription.

#### Orthopaedic surgeon questionnaire

A paper-based questionnaire was distributed at an orthopaedic trauma meeting in October 2017 and additional survey was conducted by disseminating the electronic version of the questionnaire from January to May 2018. Participants were practicing Canadian orthopaedic surgeons and participation was voluntary. The two surveys were mutually exclusive.

In the questionnaire, each surgeon was asked if they knew about the clinical use of lithium. If yes, they were asked to proceed with answering the following questions. To understand their knowledge about lithium, they were asked if lithium interacts with other drugs and if lithium has side effects. They were asked if lithium can be administered to patients with impaired renal function, and if yes, whether dose modification and extra monitoring is required. To understand their opinion on prescribing lithium for fracture healing, they were asked if they would be willing to prescribe lithium and if not, whether a pre-approved prescription protocol or scientific evidence would affect their willingness. Finally, they were asked if there was value in new treatments aimed to improve fracture healing.

## Results

### General population survey

**Fig. 1:**
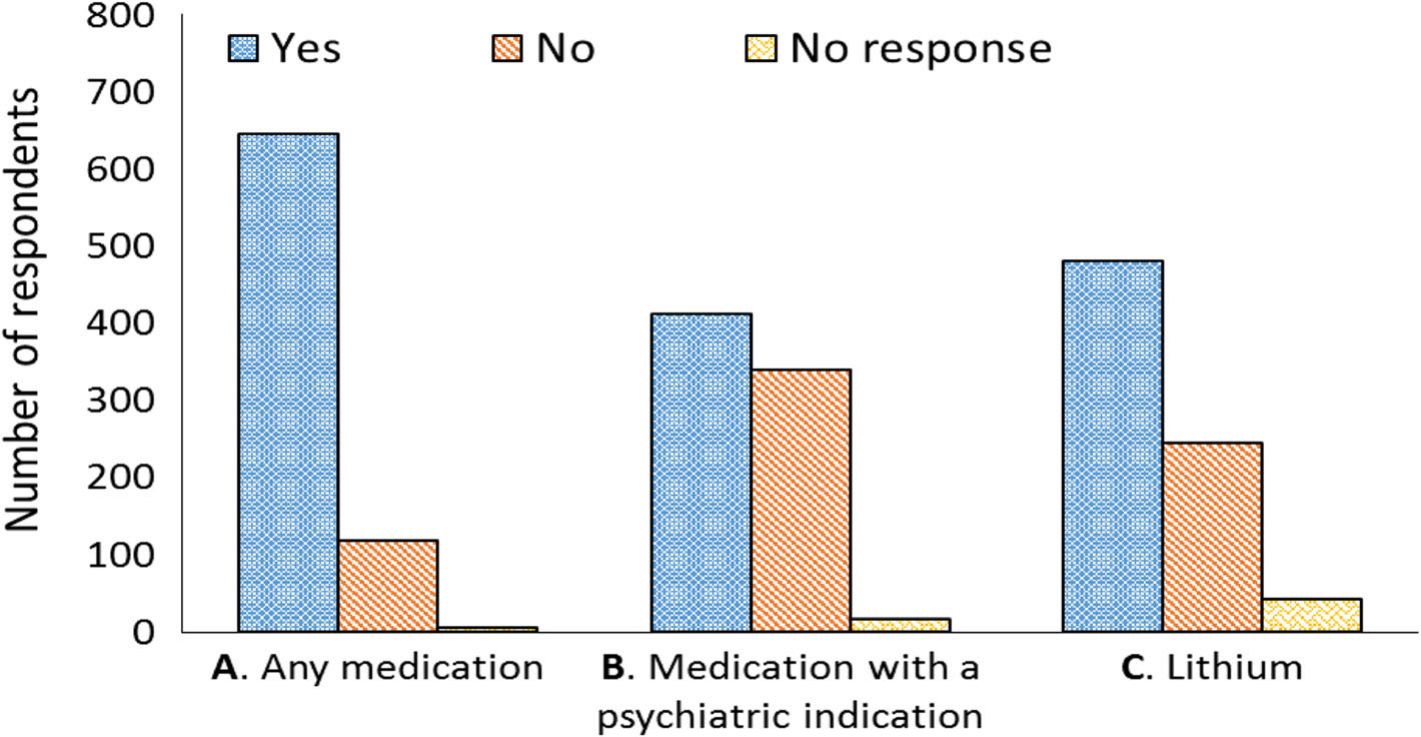
Willingness (Yes, No, No response) to take a medication (including lithium) among the general population (*n* = 768) to improve fracture healing: **a** While majority of the respondents were willing to take a medication, **b** the proportion of respondents willing to take a medication with psychiatric indication dropped. **c** A similarily lower proportion of respondents were willing to take lithium and the highest number of ‘no responses’ were noted for lithium

In total, 768 responses were collected from individuals in the Greater Toronto Area. Demographics are summarized in Table 1. Eighty four percent of survey respondents indicated they would be willing to take a medication that would aid fracture healing (Fig. 1). Only 53.6% were willing to take a medication that was also a psychiatric drug, yet 62.6% indicated they would be willing to take lithium for this purpose. The respondents were asked to justify their responses in free-text comments, as summarized in Table 2. Of those against taking a psychiatric drug for fracture healing, 27.1% said they did not want to experience side effects and 7.4% expressed a general lack of trust about using a psychiatric medication. Of those who were not willing to consider taking lithium, 30.2% were concerned about experiencing side effects and 20.4% reasoned that they did not have sufficient knowledge about lithium including its side effects to make an informed decision.

**Table 1:** Demographic characteristics of the participants in this study. Data reflects the 20/29 fracture patients who had heard of lithium and 38/43 surgeons who knew of clinical use of lithium

	N	%
General population (*N* = 768)		
Age (years)		
15–25	544	70.8
26–55	124	16.1
56+	94	12.2
No response	6	0.8
Gender		
Male	220	28.6
Female	526	68.5
Non-binary	22	2.9
Patients who did not participate in the trial (*N* = 15)		
Age (years)		
18–25	5	33.3
26–55	8	53.3
No response	2	13.3
Gender		
Male	10	66.7
Female	5	33.3
Patients who participated in the trial (*N* = 5)		
Age (years)		
18–25	0	0.0
26–55	4	80.0
No response	1	20.0
Gender		
Male	4	80.0
Female	1	20.0
Orthopaedic surgeons (*N* = 38)		
Years in practice		
< 5	11	28.9
5–15	12	31.6
> 15	14	36.8
No response	1	2.6

**Table 2:** Most common reasons stated in general population survey to justify willingness to take a medication (Including lithium) for fracture healing

Question	Response	Justification
Willing to take a medication for fracture healing	Yes (*n* = 645)	To speed healing (17.5%)
		To reduce pain (15.5%)
		No justification (43.3%)
	No (*n* = 118)	Bones can heal naturally / not necessary (31.4%)
		Do not want to experience side effects (11.9%)
		No justification (31.4%)
Willing to take a medication with psychiatric indication for fracture healing	Yes (*n* = 412)	If no side effects (15.3%)
		If prescribed (3.6%)
		No justification (45.6%)
	No (*n* = 339)	Do not want to experience side effects and/or effects on the brain (27.1%)
		Do not trust drugs (7.4%)
		No justification (40.4%)
Willing to take lithium for fracture healing	Yes (*n* = 481)	If no side effects (12.3%)
		If prescribed (8.1%)
		No justification (50.3%)
	No (*n* = 245)	Do not want to experience side effects and/or effects on the brain (30.2%)
		Lack sufficient knowledge about lithium (20.4%)
		No justification (20.8%)

Those who were willing to consider taking a drug with a psychiatric indication expressed that their willingness was contingent on experiencing minimal side effects (15.3%). Similarly, those willing to consider taking lithium in the context of fracture healing were willing if it did not have side effects (12.3%) and if it was prescribed by a physician (8.1%).

Of the 168 respondents who knew about the psychiatric use of lithium, 66.7% were willing to take a medication for fracture treatment, but only 44.6% if it was lithium. Sixty seven percent (55 respondents) were not in favor of taking lithium due to known side effects or possible effects on the brain, lack of necessity of medication for bone healing (8 respondents, 9.8%), insufficient knowledge about lithium (6 respondents, 7.3%), and skepticism about lithium’s efficacy (5 respondents, 6.1%).

A sub-group of respondents who reported having experienced a fracture (*n* = 316) was separately analyzed to assess whether their first-hand experience of the injury and the recovery process influenced their opinion on taking medications to improve fracture healing. Of these 316 respondents, 80.6% (254 respondents) were willing to take a medication to help with fracture healing. Fifty seven percent were willing to take a drug with a psychiatric indication and 64% if it was lithium. These were similar percentages to the cohort who did not report previous fractures.

### Patient survey

Thirty-one patients were eligible for the LiFT trial, and hence for the survey. Two patients could not comprehend English and hence did not participate in the survey. As such, 29 questionnaires were completed. Demographics are summarized in Table 1. Twenty participants knew about the use of lithium for clinical and/or non-clinical purposes and hence completed the rest of the questionnaire. Eleven of the 20 identified mania and/or depression as a clinical use of lithium. Five of the 20 consented to participate in the trial; this sub-group was analysed separately.

None of the 15 patients who declined to participate in the trial had taken lithium for psychiatric treatment in the past. Four (26.7%) were willing to take lithium for fracture healing. Of the 11 patients who were not willing, two indicated they would be willing if the dose was low, 2 indicated they would be willing if it was for a short duration. Of the 9 patients who identified the clinical use of lithium for mania and/or depression, 3 (33.3%) were willing to take lithium. An additional 3 patients were willing to consider lithium if the dose was low and an additional patient if it was for a short duration. Eight patients (53.3%) answered ‘Yes’ to whether lithium has side effects, while 5 (33.3%) answered ‘No’. Five patients (33.3%) indicated they would be concerned about submitting claims for lithium to their insurance company and three patients (20%) were concerned about others knowing about a lithium prescription.

Of the five patients who participated in the trial, two knew about the psychiatric use of lithium but had not previously taken it. All of the patients had graduated from a college or university (compared to only 20% of those who declined to participate in the trial). Three patients answered ‘No’ to whether lithium has side effects and two patients believed that lithium has mild side effects. None had concerns about submitting claims for lithium to their insurance company or about others knowing about a lithium prescription.

### Surgeon survey

Forty-three surveys were completed. Demographics are summarized in Table 1. Thirty-eight surgeons (88%) knew about the clinical use of lithium and as such, completed the rest of the questionnaire.

All of the surgeons correctly identified the use of lithium in psychiatric medicine. Only 11 (29%) knew that lithium interacts with other drugs, and of these, only two were able to specify these drugs (the other 27 (71%) chose ‘Don’t know’). Twenty-six of the surgeons (68%) knew that lithium has side effects, 11 (29%) did not know and one (3%) answered ‘No side effects’. Only four surgeons (11%) knew that lithium can be administered to patients with diabetes or renal impairment with dose modification and extra monitoring. Twenty-nine did not know if lithium could be administered to this patient cohort, while four respondents answered that lithium could not be administered to patients with diabetes or renal impairment.

All surgeons (*n* = 38) agreed that there is value in having new treatments for fracture healing that may reduce the number of cases of delayed healing or non-unions. Most (*n* = 34) agreed that there was value in new treatments to accelerate fracture healing in otherwise healthy patients.

Only 26 surgeons (68%) indicated they would be willing to prescribe lithium for healing fractures. Of these, 13 were willing to prescribe it independently, while 12 preferred another physician (for example, a psychiatrist) to prescribe the lithium. The remaining 12 surgeons were unsure or not willing to prescribe lithium for fracture treatment. In follow-up questions about their willingness to prescribe lithium if there was a standard protocol, 3 (of these 12) responded affirmatively, 2 responded negatively and 7 recorded no response. In assessing their willingness to prescribe lithium if there is scientific evidence demonstrating its benefit in fracture healing, 3 (of these 12) responded affirmatively, three responded negatively and 6 did not answer.

## Discussion

In considering future adoption of lithium for fracture treatment, current knowledge and perceptions of this drug highlight potential barriers to its uptake (Table 3). Knowledge about lithium may be limited in the general public, fracture patients, and orthopaedic surgeons, which impact their openness to consider its use in the context of fracture healing. Yet, there is a clear desire for new therapies to improve fracture healing.

**Table 3:** Comaprison of the three cohorts with respect to their willingness to consider using lithium for fracture healing and primary challenges to adoption of lithium

	Public	Patients	Surgeons
Percentage willing to take/prescribe lithium	62.6%	27.6%	68.0%
Barriers to adoption of lithium	- Lack of knowledge	- Lack of knowledge	- Lack of clinical knowledge
	- Concerns about side effects	- Social stigma	

Improving fracture healing through therapeutics was seen as acceptable by the majority (84%) of the general population. A stigma was found to exist with respect to taking a psychiatric drug in this context lowering the willingness to take such a medication to improve fracture healing to only 54% of respondents. Concerns about side effects, trust in psychiatric medications and lack of knowledge can all be addressed through appropriate education via clinical materials and information provided verbally by treating practitioners. Equivalent responses were found in the general public independent of whether they had previously suffered a fracture.

Those who were willing to consider taking a drug with a psychiatric indication expressed some similar concern with potential for side effects (15–17%) as well as the desire for prescription by a physician (9%). The willingness to take lithium specifically for fracture healing was lower than a non-identified psychiatric drug, primarily due to concerns over side effects or effects on the brain. Lack of a need for a medication for bone healing, insufficient knowledge about lithium and skepticism about lithium’s efficacy in the context of fracture healing were also identified. These concerns could be readily addressed through appropriate education.

The majority of long bone fracture patients seen at Sunnybrook Health Science Centre (69%) had heard of lithium, with 38% aware of its clinical use in psychiatry (as compared to the 22% in the general population survey). Of the 20 patients completing the survey, 40% were willing to take lithium for fracture healing. Low dose and/or short duration of use increased those willing by 10%. Half the patients acknowledged that lithium has side effects; education as to possible side effects at the lower dose for fracture healing would be critical information for patients considering this therapeutic. Social stigma related to lithium was identified with concerns about submitting insurance claims (33%) and others knowing about a lithium prescription (20%) among those who declined to participate in the LiFT trial. If lithium therapy for fracture healing becomes a standard of care, such barriers may shrink, but remain substantial in considering early adoption.

New treatments for fracture healing are clearly desired by orthopaedic surgeons, specifically those that may reduce the number of cases of delayed healing or non-unions. The acceleration of fracture healing in otherwise healthy patients was also deemed important by ~ 90% of the responding surgeons. As such there is an opportunity for new therapeutics in both contexts from a clinical perspective, suggesting a potential for widespread utilization of a new or repurposed drug or therapy to enhance fracture healing.

There is a lack of knowledge about lithium in the orthopaedic surgeon community, despite most respondents knowing of its use in psychiatric medicine (88%). Dissemination of accurate knowledge about lithium including dose dependent side effects and precautions for use in patients with diabetes or impaired renal function is needed if lithium is to be considered for clinical use in the context of fracture healing. Initial uptake may be improved if another experienced physician can be involved in the prescription of the lithium. A standard protocol would likely only have a modest initial effect in improving some surgeons’ willingness to prescribe lithium but may be worthwhile to ensure proper and consistent implementation. While drug interactions may pose less of a barrier in the treatment of otherwise healthy individuals with a long bone fracture, the use of lithium in the context of osteoporotic fracture healing may require more detailed knowledge and the dissemination of this knowledge to multiple practitioners involved in a patient’s circle of care. Adoption planning must consider the current limited knowledge state and address this gap through information dissemination and training, perhaps initially with interdisciplinary mentorship support (i.e. from psychiatry). Overcoming such adoption barriers in the context of lithium for fracture healing may provide an important broader context to enable uptake of this and other repurposed drugs/psychiatric drugs for new indications.

Surprisingly, 17% of orthopaedic surgeon respondents who had heard of lithium were unsure or unwilling to consider its use in the context of fracture healing even if there were scientific evidence demonstrating its benefit. While this could be due to the perception that in the short term most fractures will heal, the unanimously identified need for new treatments for fracture healing suggest that perhaps this is not the case. Differences in early vs. late adopters of new therapeutics may explain some of the variability in the responses of surgeons. As such, uptake in lithium usage for fracture healing may initially follow a limited incremental adoption model. Randomized controlled data (level 1 evidence) followed by longer term clinical experience from early adopters may be required before it can be more widely accepted as a possible standard of care.

Although this study provides crucial information for secondary adoption of lithium, it has limitations. Firstly, the group sizes for patient and surgeon surveys were small. While the volume of trauma patients with fractures received at the site hospital was considerable, only a small proportion of these patients were eligible for the LiFT trial, and hence for the survey due to the strict inclusion and exclusion criteria intended for a more homogenous healthy patient cohort. For the surgeon survey, the low recruitment was attributed to the voluntary nature of their participation. Secondly, those patients who accepted to participate in the trial took the survey at their final visit (6–10 weeks post-acceptance) as opposed to those patients who declined to participate in the trial and took the survey immediately after making this decision. The difference in timing was mainly for logistical reasons for the trial recruitment team. However, regardless of the timing, all the patients were asked to fill the questionnaire based on their knowledge and perception of lithium prior to learning about the trial to minimize the bias in their responses.

## Conclusions

This study identified a lack of knowledge about indications, side effects and dosage of lithium in the general population, patients with long bone fractures and orthopaedic surgeons that have the potential to delay early adoption, even with strong clinical evidence. Development of a robust educational framework for orthopaedic surgeons, their patients and the members of their clinical care teams will be essential to translation. Ultimately, the success of lithium in the context of fracture healing with early adopters will be needed to drive widespread acceptance of repurposing of this drug into orthopaedic fracture care.

## Additional file

Additional file 1: A. General public questionnaire. B. Patient questionnaire. C. Orthopaedic surgeon questionnaire. (DOCX 16 kb)

## Data Availability

The datasets used and/or analysed during the current study are available from Dr. Diane Nam on reasonable request.

## Abbreviations

GSK-3β: Glycogen synthase kinase-3β
LiFT: Lithium for fracture treatment
Wnt: Wingless
