# Supplementary material for: Perceptions of using lithium in fracture management: a survey of orthopaedic surgeons, fracture patients and the general public
Source: BMC Musculoskelet Disord. 2019 Aug 31;20:389. doi: 10.1186/s12891-019-2772-0 (PMC6717343; doi:10.1186/s12891-019-2772-0)
Supplement: Supplementary file 1 — A. General public questionnaire. B. Patient questionnaire. C. Orthopaedic surgeon questionnaire. (DOCX 16 kb) [file 12891_2019_2772_MOESM1_ESM.docx]

| **Demographic information** |
| --- |
| 1. Gender Male___ Female___ Non-binary___ Prefer not to answer___ |
| 1. Year of birth: |
| 1. Occupation: |
| **Knowledge about fracture and lithium drug** |
| 1. Do you have any conditions that puts you at a higher risk of bone fractures (ex. Osteoporosis, Osteopenia, etc) Yes___ No___ |
| 1. Have you ever broken or fractured a bone? Yes___ No___   If yes, when? (provide year(s))  If yes, did you take any medication(s) as part of your bone break or fracture treatment? Yes___ No___  If yes, what medication(s)? |
| 1. Lithium is a prescribed medication. Do you know what it can be used to treat? Yes___ No___   If yes, please list its use(s). |
| **Opinion on use of lithium drug for fracture healing** |
| 1. If you were to break or fracture a bone in the future, would you consider taking medication to help with healing? Yes___ No___   Why/why not: |
| 1. Lithium can help heal bones. Would you take it to help with healing after a bone break or fracture? Yes___ No___   Why/why not: |
| 1. Would you be willing to take a medication that is also prescribed for treating mental illnesses to help with healing after a bone break or fracture? Yes___ No___   Why/why not: |

**Additional file 1.A. General public questionnaire.**

| 1. Please enter your study ID below. ___ |
| --- |
| 1. Please write in today’s date. ___ |
| 1. Please write the year you were born. ___ |
| 1. Are you here because of a broken bone? No *This is the end of the survey for you, thank you for your time*___ Yes *Please continue*___ |
| 1. Which bone(s) did you break? Refer to the image below for guidance. |
| 1. Are you: Male___ Female___ Other___ Prefer not to answer___ |
| 1. What is the highest degree or level of school you have completed (if currently enrolled, highest degree received)?   Less than high school___  High school graduate, diploma, or the equivalent (for example: GED)___  Some college/university (no degree)___  Trade/technical/vocational training___  Graduated from college/university___  Postgraduate school or degree___  Prefer not to answer___ |
| 1. Have you ever heard of Lithium? No *This is the end of the survey for you, thank you for your time*___ Yes *Please continue*___ |
| 1. What is Lithium used for?   Making batteries___  Heart problems___  Depression or other mental troubles___  Cancer___ |
| 1. Have you ever taken Lithium? Yes___ No___ Prefer not to answer___ |
| 1. If you have taken Lithium, do you still take it now? Yes___ No___ Not applicable___ |
| 1. If you have taken Lithium but no longer do, why did you stop? Side effects___ Stigma___ No longer needed___ Not applicable___ Other (please specify)___ |
| 1. Would you be willing to take Lithium to improve your bone healing? Yes___ No___ |
| 1. Would a low dose of Lithium affect your willingness to take it? Yes___ No___ |
| 1. Would a short duration of treatment affect your willingness to take Lithium? Yes___ No___ |
| 1. Do you think Lithium has side effects? Yes___ No___ |
| 1. If you answered Yes to ‘Do you think Lithium has side effects’, are there many side effects? Yes___ No___ Not applicable___ Comment___ |
| 1. If you answered Yes to ‘Do you think Lithium has side effects’, are the side effects: Mild__ Moderate___ Severe___ Not applicable___ Comment___ |
| 1. Would you have any concerns in submitting a drug claim for Lithium to your insurance company? Yes___ No___ Comment___ |
| 1. Do you have any concerns about your family, friends or employer knowing you have been prescribed Lithium? Yes___ No___ |
| 1. Do you have any other concerns about the use of Lithium in bone healing? Comment___ |

**Additional file 1.B. Patient questionnaire.**

| 1. Please write in today’s date. ___ |
| --- |
| 1. Number of years that you have been practicing orthopaedic surgery: <5___ 5-15___ >15___ |
| 1. Have you heard of using Lithium in a clinical context (for any reason)? No *This is the end of the survey- Thank you for your time*___ Yes *Please continue*___ |
| 1. What clinical areas is Lithium used for? Cardiac issues___ Depression___ Mania___ Cancer___ Stroke___ ALS___ I don’t know___ Other (please specify)___ |
| 1. Does Lithium have interactions with other drugs? No___ Don’t know___ Yes___ If Yes, please specify___ |
| 1. Does Lithium have side effects/toxicity? No___ Don’t know___ Yes___   If Yes:   1. Please choose the side effects that apply: Renal dysfunction___ Diarrhea___ Nausea___ Dry mouth___ Thirst___ Weakness___ 2. Are the side effects dose dependent? Yes___ No___ |
| 1. Can Lithium be used in patients with diabetes or renal impairment? No___ Don’t know___ Yes___   If Yes:   1. If administered to patients with diabetes or renal impairment, are modifications to Lithium dosing required? No___ Don’t know___ Yes___ 2. If administered to patients with diabetes or renal impairment, is extra monitoring required? No___ Don’t know___ Yes___ |
| 1. Would you be willing to prescribe Lithium if there was evidence to show it to be of significant benefit in fracture healing? No___ Don’t know___ Yes___   If Yes to Q12:   1. Would you be comfortable prescribing Lithium or would you need someone else to prescribe it? Yes (I can prescribe)___ No (Prefer someone else to prescribe)___   If No to Q12:   1. Would you feel more comfortable prescribing Lithium if there was a set protocol with a standard order sheet that has been pre-approved for indication in the fracture population? Yes___ No___ Don’t know___ 2. Even if there was level 1 scientific data supported Lithium’s efficacy in fracture healing, would you still have reservations using Lithium in your practice? Yes___ No___ Don’t know___ |
| 1. Do you see a value in new treatments to accelerate acute fracture healing in otherwise healthy adults? No___ Don’t know___ Yes___ |
| 1. Do you see a value in new treatments to reduce delayed/non-union in otherwise healthy adults? No___ Don’t know___ Yes___ |
| 1. Would you be interested in learning more about the role of Lithium in improved fracture healing? No___ Don’t know___ Yes___ |

**Additional file 1.C. Orthopaedic surgeon questionnaire.**
